# ABO-Incompatible Kidney Transplantation

**DOI:** 10.3389/fimmu.2017.00234

**Published:** 2017-03-06

**Authors:** Christian Morath, Martin Zeier, Bernd Döhler, Gerhard Opelz, Caner Süsal

**Affiliations:** ^1^Department of Nephrology, University of Heidelberg, Heidelberg, Germany; ^2^Transplantation Immunology, Institute of Immunology, University of Heidelberg, Heidelberg, Germany

**Keywords:** kidney transplantation, ABO incompatible, survival, desensitization, antibodies

## Abstract

ABO-incompatible (ABOi) kidney transplantation has long been considered a contraindication to successful kidney transplantation. During the last 25 years, increasing organ shortage enforced the development of strategies to overcome the ABO antibody barrier. In the meantime, ABOi kidney transplantation has become a routine procedure with death-censored graft survival rates comparable to the rates in compatible transplantations. Desensitization is usually achieved by apheresis and B cell-depleting therapies that are accompanied by powerful immunosuppression. Anti-A/B antibodies are aimed to be below a certain threshold at the time of ABOi kidney transplantation and during the first 2 weeks after surgery. Thereafter, even a rebound of anti-A/B antibodies does not appear to harm the kidney transplant, a phenomenon that is called accommodation, but is poorly understood. There is still concern, however, that infectious complications such as viral disease, *Pneumocystis jirovecii* pneumonia, and severe urinary tract infections are increased after ABOi transplantations. Recent data from the Collaborative Transplant Study show that during the first year after kidney transplantation, one additional patient death from an infectious complication occurs in 100 ABOi kidney transplant recipients. Herein, we review the recent evidence on ABOi kidney transplantation with a focus on desensitization strategies and respective outcomes.

## Introduction

In an earlier publication from 1955, Hume et al. expressed their skepticism on the feasibility of ABO-incompatible (ABOi) kidney transplantations: “… *we do not feel that renal transplantation in the presence of blood incompatibility is wise*” (1). Since then, major ABO incompatibility has been considered a contraindication to kidney transplantation. A major breakthrough came in 1982, with the first large study on ABOi kidney transplantation by Alexandre et al. from Belgium (2, 3). Successful desensitization was achieved by repeated plasmapheresis (PP), splenectomy, donor thrombocyte transfusion, and infusion of A or B trisaccharide, together with intensified immunosuppression. One-year graft survival in this study was a remarkable 75%. This led to a wider utilization of ABOi kidney transplantations, first in Japan from the late 1980s, in the US from the mid 1990s, and in Europe from the early 2000s. While, even today, kidney transplantation is best performed in the absence of (major) ABO incompatibility, a large end-stage kidney disease population and an increasing organ shortage result in waiting times for a deceased donor kidney transplant exceeding 5 years in some countries such as Germany. One possibility to reduce the waiting time is the transplantation across ABO antibody barriers. Theoretically, the number of kidney transplantations from living donors can be increased by up to 30% when patients are transplanted across the ABO antibody barrier. With currently existing protocols, as many as 90% of patients with an ABOi living donor may effectively be desensitized and transplanted. The aim of desensitization protocols is the reduction and maintenance of anti-A/B antibodies (isoagglutinins) during the first 2 weeks after transplantation below a threshold that is considered to be safe (e.g., <1:32 in tube technique). Thereafter, even when anti-A/B antibodies recur at high levels they will not harm the kidney transplant, a phenomenon that is called accommodation. In recent years, graft survival rates after ABOi kidney transplantation nearly equaled those after ABO-compatible (ABOc) procedures.

Herein, we review the latest efforts and results in kidney transplantation across the ABO antibody barrier.

## Blood Group Antigens and Antibodies

The ABO antigen system consists of oligosaccharides that are predominantly expressed on red blood cells and are also found on endothelial cells, tubuli, and glomeruli making the ABO antigen system important for kidney transplantation. Patients with different blood groups differ with respect to their antigen density on erythrocytes. Compared to blood group A1 and blood group B individuals, blood group A2 recipients, who make up 20% of all Caucasian individuals with blood group A, have a low expression of blood group antigen molecules (30–50%) on the surface of erythrocytes, which is believed to be responsible for the lower immunogenicity of organs from blood group A2 donors (4, 5). ABOi kidney transplantation with A2 organs has been accomplished with standard immunosuppressive therapy without any additional measures (6). Of interest, anti-A/B antibodies are formed upon contact with gut bacteria during early infancy. Naturally occurring anti-A/B antibodies are predominantly of the IgM class but especially in blood group O individuals they also consist of IgG and IgA class (7). While the pathogenic importance of anti-A/B antibodies in solid organ transplantation is well known, the relative contribution of the different immunoglobulin isotypes and their subclasses to organ rejection remains to be elucidated.

## Desensitization for ABOi Kidney Transplantation

**Figure 1:**
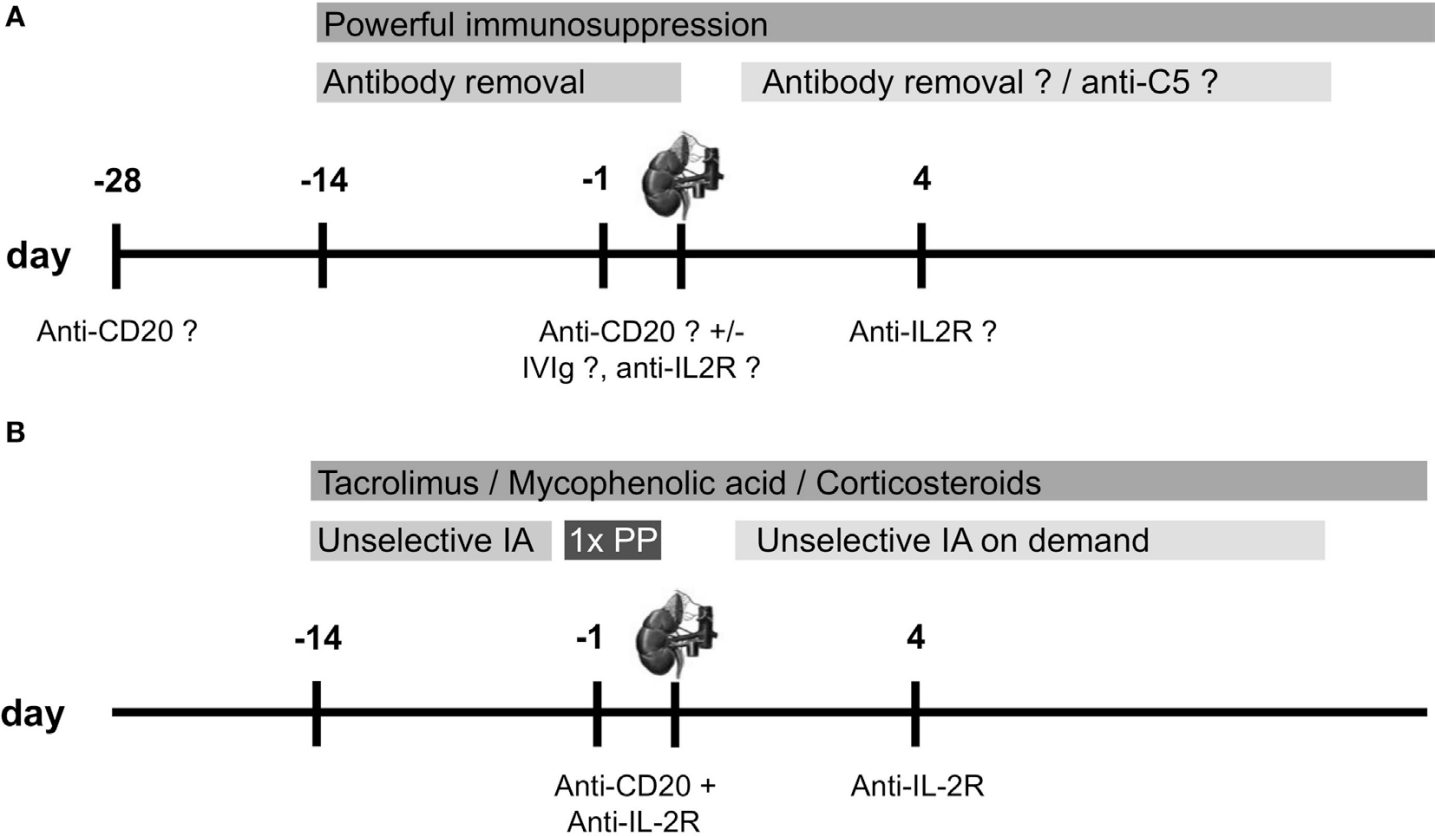
**Overview of desensitization protocols for ABO-incompatible (ABOi) living donor kidney transplantation**. **(A)** Scheme for a standard desensitization protocol performed by the majority of centers with modifications in the utilization of desensitization devices, and **(B)** desensitization protocol for ABOi living donor kidney transplantation at the University of Heidelberg. Anti-CD20 therapy is usually performed with rituximab 375 mg/m^2^, anti-IL-2R therapy is performed with basiliximab 20 mg [modified from Ref. (8)]. IA, immunoadsorption; IVIg, intravenous immunoglobulin; PP, plasmapheresis.

Despite the absence of a generally accepted desensitization protocol for the transplantation across the ABO antibody barrier, all currently proposed strategies share some common principles (Figure 1A).

These include together with a powerful maintenance immunosuppression one or more of the following:

(1) Anti-A/B antibody depletion at the time of transplantation using PP, double-filtration PP/membrane filtration, or selective or unselective immunoadsorption (IA)
(2) Modulation of the recipient’s immune system by the use of intravenous immunoglobulins (IVIgs)
(3) Reduction of the B lymphocyte pool by splenectomy, or more recently by the anti-CD20 antibody rituximab
(4) Prevention of the deleterious consequences of complement activation upon anti-A/B antibody binding to the graft endothelium.

### Antibody Depletion by Extracorporal Treatment

Antibody removal strategies may be divided into methods that completely remove plasma proteins such as PP, methods that remove only a specific fraction of the plasma proteins including the immunoglobulins (such as membrane separation), and more specific methods such as unselective or selective IA. While PP is the preferred antibody removal strategy in the US, membrane separation is popular in Japan. Unselective and selective IAs are often used in Europe.

Selective anti-A/B antibody removal is feasible by the Glycosorb columns that contain synthetic terminal trisaccharide A or B blood group antigen linked to a sepharose matrix. In addition, they may also reduce total IgG as well as IgG against polysaccharide antigens such as anti-*Pneumococcus* IgG (9). In a recent analysis, Wahrmann et al. found single treatments with unselective IA to be more effective than with selective anti-A/B antibody columns in removing anti-A/B IgG (median reduction to 28 versus 59% of baseline, *P* < 0.001) (10). In contrast, unselective IA was less effective in the removal of anti-A/B antibodies of the IgM (74 versus 30%, *P* < 0.001) and IgG3 subclass (72 versus 42%, *P* < 0.05). The same group found that a combination of membrane separation and unselective IA effectively removed IgG and IgM antibodies and effector molecules such as complement C1q component (11). No significant differences were found in clinical studies that compared the impact of different IA strategies on clinical outcomes, including anti-A/B antibody reduction, survival, kidney function, rejection episodes, or complications (12).

### Intravenous Immunoglobulins

Intravenous immunoglobulins are given by many centers before ABOi kidney transplantation to prevent the anti-A/B antibody rebound in the early phase after transplantation. In addition, IVIg infusion is believed to reduce infectious complications by substituting depleted immunoglobulins. As a note of caution, IVIg preparations contain IgG antibodies directed against A/B antigens and can effectively increase anti-A/B antibody titers upon administration (12, 13).

### B-Cell Depletion by Splenectomy or Rituximab

Before the introduction of pharmacological anti-B cell therapies, splenectomy was an integral component for the reduction of the B lymphocyte pool prior to ABOi kidney transplantation. Due to the surgical risk and increased risk of sepsis, splenectomy was gradually substituted by the anti-CD20 antibody rituximab. More recently, several groups completely abandoned anti-B cell therapies from their protocols. Flint et al. reported on 37 patients from Melbourne who underwent ABOi kidney transplantation after antibody removal by PP (14). Transplantation was accomplished by the use of standard immunosuppressive therapy without rituximab when the patient had reached an anti-A/B antibody titer of less than 1:32 (tube method). Patient and graft survival in this cohort was 100% after a median of 26 months after transplantation. Two antibody-mediated rejection episodes were successfully reversed. We observed in the Collaborative Transplant Study (CTS) a higher rate of death-censored graft loss in ABOi kidney transplant recipients when rituximab was omitted (see below) (15).

### Inhibition of Complement Activation

An emerging new concept in the transplantation across ABO antibody barriers is the inhibition of complement activation upon binding of antibodies to the allograft endothelium. Biglarnia et al. described an intentional simultaneous ABOi kidney pancreas transplantation (16). Severe antibody-mediated rejection in this patient during anti-A/B antibody rebound was successfully treated by inhibiting the assembly of the membrane attack complex by eculizumab. Stegall et al. performed a single center study to evaluate the efficacy of eculizumab added to conventional therapy in the prevention of antibody-mediated rejection after ABOi living donor kidney transplantation (unpublished, NCT01095887). Patients received eculizumab at the time of transplantation, on day 1 after surgery and weekly thereafter for 4 weeks. The study was terminated after only six patients due to poor enrollment. Two of the six patients reached the primary study endpoint of antibody-mediated rejection after 3 months and two allografts had to be removed during the study period. Therefore, results on the use of eculizumab after ABOi kidney transplantation are inconclusive.

## Desensitization Protocols and Survival after ABOi Living Donor Kidney Transplantation

Table S1 in Supplementary Material gives an overview over studies on ABOi kidney transplantation. The largest cohort of patients after ABOi kidney transplantation with the longest follow-up of more than 20 years is reported from Japan. Most patients were desensitized by double-filtration PP, and splenectomy was more recently replaced by the anti-CD20 antibody rituximab. For the most recent area from 2001 to 2010, patient and graft survival rates for the 1,427 analyzed patients were an excellent 98 and 96% for the first year, and 91 and 83% after 9 years, respectively (17). Data from the Scientific Registry of Transplant Recipients on the outcomes of 738 ABOi kidney transplantations that were performed between 1995 and 2010 in the US have recently been published (18). Most patients were desensitized by PP and low-dose IVIg. The cumulative incidence of graft loss during the first year after transplantation was 5.9% in ABOi as compared to only 2.9% in ABOc transplantations and occurred mainly during the first 2 weeks after surgery due to rejection. In 2003, Tydén et al. from Sweden published a protocol for ABOi transplantation that is based on recipient desensitization by selective IA using Glycosorb columns (19). In addition, splenectomy was replaced by the anti-CD20 antibody rituximab. This protocol led to a renaissance of ABOi kidney transplantation in Europe. Recently, Genberg et al. published their extended experience with this protocol (20). Of 45 patients desensitized for ABOi kidney transplantation, 43 were eventually transplanted between September 2001 and May 2010 (96%). Overall patient and graft survival after a mean follow-up of 4.5 years was 93 and 91%, respectively. None of the patients experienced early acute antibody-mediated rejection that could be linked to anti-A/B antibodies. Recently, the Freiburg group from Germany compared the results of 100 ABOi kidney transplantations performed between April 1, 2004, and October 28, 2014, with the results of 248 ABOc transplantations performed during the same time period (21). Using the Stockholm protocol, they achieved in recipients of ABOi transplants a 10-year patient and death-censored graft survival of 99 and 94%, respectively, which did not differ significantly from the 80 and 88% survival rates, respectively, in recipients of ABOc transplants. The rates for antibody- and T-cell-mediated rejections were also not significantly different. A study from the UK showed similar death-censored graft survival in 62 patients 3 years after ABOi kidney transplantation when compared to ABOc controls (22). However, patient survival in ABOi transplant recipients was reduced due to infectious complications, mostly *Pneumocystis jirovecii* pneumonia.

Since 2006, at our center in Heidelberg, we have been using a protocol for desensitization of ABOi kidney transplant candidates that is very similar to the Swedish protocol (Figure 1B) (8, 12). The major difference is the use of unselective instead of selective IA, allowing also the desensitization for HLA-incompatible living donor kidney transplantation. Further differences are the omission of IVIg application and a variable number of IA treatments depending on the strength of anti-A/B antibody. To remove pathogenically relevant anti-A/B antibodies of the IgM class more efficiently, at least one additional PP treatment was performed in all patients the day before surgery as of August 2012 (23). An early analysis of ABOi kidney transplantations showed successful desensitization of 12 patients after a median of six IA treatments (12). Anti-A/B titer reduction with unselective IA was comparable to that of a historical control group that received selective IA. In a more recent analysis, we compared 34 ABOi kidney transplant recipients who were desensitized with unselective IA to 68 matched, standard risk living donor kidney recipients (23). After a median postoperative follow-up of 22 months, graft survival in ABOi kidney transplant recipients was insignificantly lower compared to standard risk recipients (*P* = 0.05). One of the two patient deaths in the ABOi kidney transplant recipients was due to *P. jirovecii* pneumonia at postoperative day 169. This patient death may be attributable to intensified immunosuppression that was applied during desensitization including rituximab. Other important differences between ABOi and standard risk kidney recipients were a higher incidence of BK virus replication (>10^4^ copies/mL plasma, 21 versus 6%, *P* = 0.04) and BK virus nephropathy (SV 40 positive in biopsy, 12 versus 0%, *P* = 0.01) and a higher prevalence of colonization with multidrug-resistant bacteria (15 versus 1%, *P* = 0.02).

## Results from the CTS

**Figure 2:**
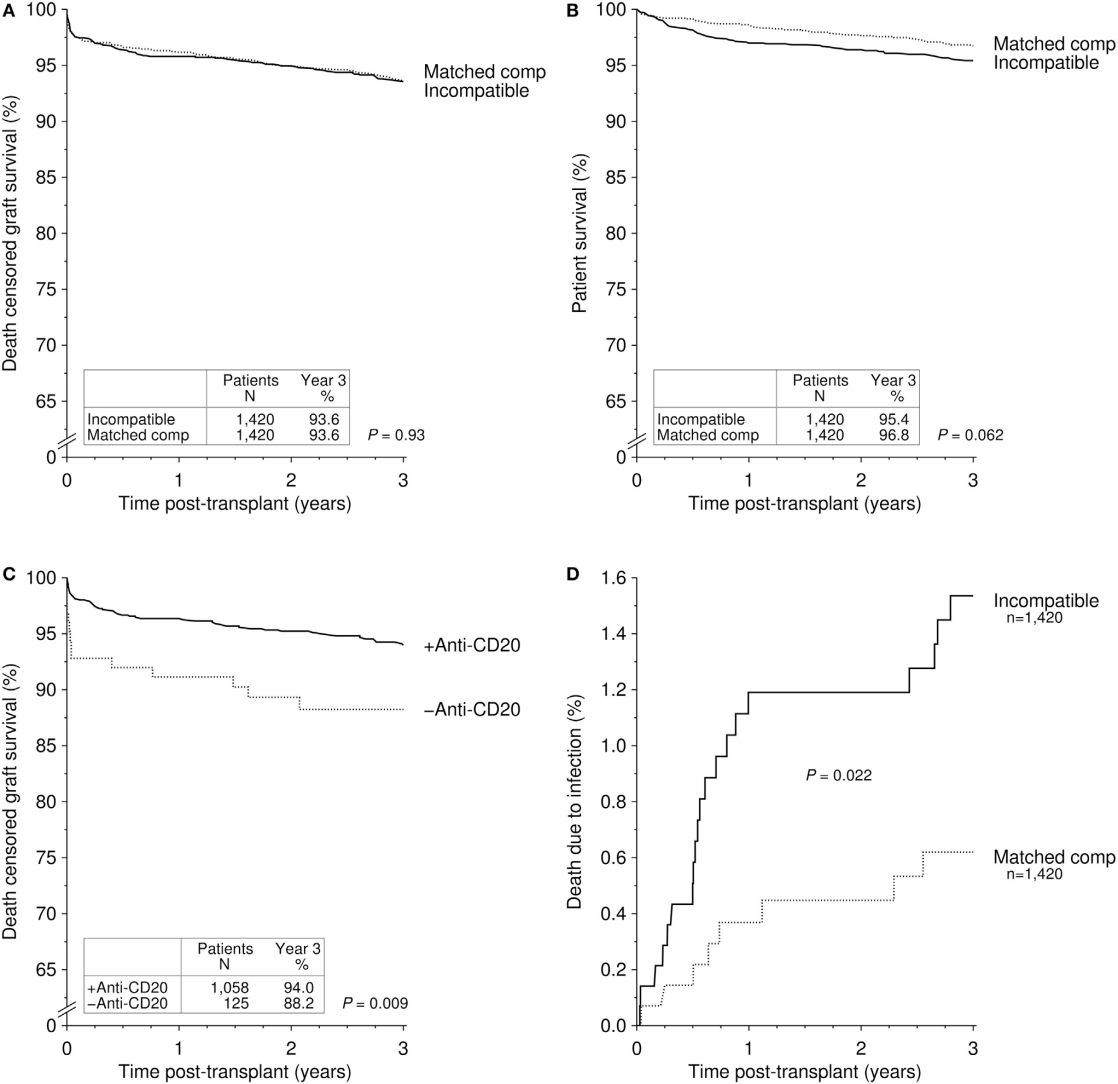
**Cumulative incidence of (A) death-censored graft survival and (B) patient survival in recipients of an ABO-incompatible (ABOi) living donor graft and matched controls receiving an ABO-compatible (ABOc) living donor graft [updated Figure 1 of Ref. (15)]**. **(C)** Cumulative incidence of death-censored graft survival in recipients of an ABOi living donor graft with and without anti-CD20 antibody treatment [updated Figure 4 of Ref. (15)]. **(D)** Cumulative incidence of death due to infection in recipients of an ABOi living donor graft and matched controls receiving an ABOc living donor graft [updated Figure 3 of Ref. (15)].

We recently published 3-year outcomes of 1,420 ABOi kidney transplant recipients who were transplanted at 101 different centers between 2005 and 2012 (15). Patients were compared to a matched group of ABOc kidney transplant recipients and to all ABOc kidney transplant recipients from centers that performed at least five ABOi procedures. Overall graft, death-censored graft, and patient survival were not statistically significant different between the groups. Early patient survival was reduced in ABOi kidney transplant recipients due to a higher rate of early infection-associated death (*P* = 0.037 versus matched controls and *P* < 0.001 versus center controls). Specifically, one additional death per 100 patients occurred in the first year after ABOi kidney transplantation from an infectious complication. Figure 2 provides the updated results for this cohort of 1,420 ABOi kidney transplant recipients (15). Of note, a trend toward better 3-year death-censored graft survival in patients receiving anti-CD20 therapy (*P* = 0.081) in the meantime has become statistically significant after longer follow-up (*P* = 0.009, Figure 2C), suggesting a need for anti-B cell therapies in the setting of ABO incompatibility.

## Complications and Hurdles of ABOi Kidney Transplantation

### Accomodation versus Rejection

In contrast to transplantation in the HLA-sensitized patient, accommodation appears to be a frequent phenomenon after ABOi kidney transplantations and is often associated with C4d deposition in peritubular capillaries of allograft biopsies. An accommodation phenotype may be achieved by the controlled anti-A/B antibody exposure to antigens in the early phase after kidney transplantation. About 2 weeks after successful transplantation, accommodation is established and even high anti-A/B antibody exposure does not harm the kidney transplant. Local upregulation of complement regulatory proteins, like CD45, CD55, and CD59, as a consequence of anti-A/B antibody-dependent inactivation of ERK1/2 signaling pathway are discussed as one possible mechanism (24).

### Infection and Malignancy

There are conflicting results on infectious complications after ABOi kidney transplantation in the literature. A higher frequency of viral infections such as CMV, HSV, VZV, and BK virus, as well as *P. jirovecii* pneumonia, wound, and severe urinary tract infections have been described (22, 25, 26). In the CTS and the Heidelberg cohort, an increased risk for early severe infections was observed, resulting in approximately one additional patient death in 100 ABOi kidney transplant recipients during the first year after surgery (15, 23). We and others also observed a higher incidence of BK virus replication and BK virus-associated nephropathy (23). Of note, in a study by Sharif et al., the rate of BK virus nephropathy was about three times higher in ABOi patients compared to patients with HLA antibodies, despite comparable immunosuppressive therapy (27). Bentall et al. hypothesized that different blood group antigens may influence binding of viral pathogen receptors to sialic acid on renal tubular cells (28).

Hall et al. found no increased cancer risk when comparing 318 ABOi kidney transplant recipients to matched ABOc controls (29). The analysis of 1,420 ABOi transplantations from the CTS study also did not show an increased risk of malignancy in ABOi compared to ABOc patients (15).

### Additional Observations

A study from the US Renal Data System registry found a two times higher risk of early hemorrhage in 119 ABOi kidney transplant recipients when compared to ABOc controls (adjusted HR, 1.96, *P* < 0.05) (26). A higher bleeding risk was also found in a cohort of pediatric kidney transplant recipients with two major bleeding episodes in three patients, which was attributed to the unspecific binding of coagulation factors during repeated IA (30). This assumption is supported by the findings of de Weerd et al. who found a significant correlation between the number of pretransplant apheresis treatments and the peri- and posttransplant bleeding risk (31).

Some authors observed an increased rate of surgical complications after ABOi kidney transplantation, which were attributed to early intensified immunosuppression with mycophenolic acid and removal of coagulation factors by apheresis. The Freiburg group reported a significantly higher number of lymphoceles in ABOi patients than in ABOc controls (33 versus 15%; *P* = 0.003) that required surgical revisions in 20 and 8% (*P* = 0.013) of patients, respectively (21). Also, the overall need for surgical revision was significantly higher in ABOi patients compared to ABOc controls (38 versus 24%, *P* = 0.032).

## Future Perspectives

A new strategy that may come into clinics in the future is the reduction of blood group antigen levels in the allograft by *ex vivo* infusion of endo-beta-galactosidase (32).

Another approach is the complete avoidance of the ABO antibody barrier by kidney exchange programs. However, despite the usage of large kidney exchange programs, including the utilization of altruistic donors, the blood group O recipients accumulated on the waiting list in different studies (33). Desensitization for ABOi kidney transplantation was the only way to transplant these patients within a reasonable period of time.

## Conclusion

In recent years, ABOi kidney transplantation has become a routine procedure. By this approach, about 30% of living donors who were refused in the past can now donate their kidneys and thereby significantly expand the living donor pool. Transplantation in the presence of major ABO incompatibility, however, places the patient at a somewhat higher risk of early rejection, infection, and infection-associated death. Therefore, whenever possible, ABOc procedures should be preferred.

## Author Contributions

All authors substantially contributed to preparation of the manuscript and gave final approval for publication.

## Conflict of Interest Statement

The authors declare that the research was conducted in the absence of any commercial or financial relationships that could be construed as a potential conflict of interest.

## References

[1] Hume DM Merrill JP Miller BF Thorn GW Experiences with renal homotransplantation in the human: report of nine cases. J Clin Invest (1955) 34(2):327–82. 10.1172/JCI103085 13233354 PMC438633

[2] Alexandre GP De Bruyere M Squifflet JP Moriau M Latinne D Pirson Y Human ABO-incompatible living donor renal homografts. Neth J Med (1985) 28(6):231–4. 3892322

[3] Squifflet JP De Meyer M Malaise J Latinne D Pirson Y Alexandre GP. Lessons learned from ABO-incompatible living donor kidney transplantation: 20 years later. Exp Clin Transplant (2004) 2(1):208–13. 15859930

[4] Rydberg L Breimer ME Samuelsson BE Brynger H Blood group ABO-incompatible (A2 to O) kidney transplantation in human subjects: a clinical, serologic, and biochemical approach. Transplant Proc (1987) 19(6):4528–37. 2447692

[5] Rydberg L Breimer ME Brynger H Samuelsson BE ABO-incompatible kidney transplantation (A2 to O). Qualitative and semiquantitative studies of the humoral immune response against different blood group A antigens. Transplantation (1990) 49(5):954–60. 10.1097/00007890-199005000-00023 2336711

[6] Forbes RC Feurer ID Shaffer D. A2 incompatible kidney transplantation does not adversely affect graft or patient survival. Clin Transplant (2016) 30(5):589–97. 10.1111/ctr.12724 26913566

[7] Rieben R Buchs JP Fluckiger E Nydegger UE. Antibodies to histo-blood group substances A and B: agglutination titers, Ig class, and IgG subclasses in healthy persons of different age categories. Transfusion (1991) 31(7):607–15. 10.1046/j.1537-2995.1991.31791368336.x 1891791

[8] Becker LE Susal C Morath C. Kidney transplantation across HLA and ABO antibody barriers. Curr Opin Organ Transplant (2013) 18(4):445–54. 10.1097/MOT.0b013e3283636c20 23838650

[9] Valli PV Puga Yung G Fehr T Schulz-Huotari C Kaup N Gungor T Changes of circulating antibody levels induced by ABO antibody adsorption for ABO-incompatible kidney transplantation. Am J Transplant (2009) 9(5):1072–80. 10.1111/j.1600-6143.2009.02579.x 19344433

[10] Wahrmann M Schiemann M Marinova L Kormoczi GF Derfler K Fehr T Anti-A/B antibody depletion by semiselective versus ABO blood group-specific immunoadsorption. Nephrol Dial Transplant (2012) 27(5):2122–9. 10.1093/ndt/gfr610 22086972

[11] Eskandary F Wahrmann M Biesenbach P Sandurkov C Konig F Schwaiger E ABO antibody and complement depletion by immunoadsorption combined with membrane filtration – a randomized, controlled, cross-over trial. Nephrol Dial Transplant (2014) 29(3):706–14. 10.1093/ndt/gft502 24378525

[12] Morath C Becker LE Leo A Beimler J Klein K Seckinger J ABO-incompatible kidney transplantation enabled by non-antigen-specific immunoadsorption. Transplantation (2012) 93(8):827–34. 10.1097/TP.0b013e31824836ae 22382504

[13] Staley EM Carruba SS Manning M Pham HP Williams LA III Marques MB Anti-blood group antibodies in intravenous immunoglobulin may complicate interpretation of antibody titers in ABO-incompatible transplantation. Am J Transplant (2016) 16(8):2483–6. 10.1111/ajt.13760 26913485

[14] Flint SM Walker RG Hogan C Haeusler MN Robertson A Francis DM Successful ABO-incompatible kidney transplantation with antibody removal and standard immunosuppression. Am J Transplant (2011) 11(5):1016–24. 10.1111/j.1600-6143.2011.03464.x 21449947

[15] Opelz G Morath C Susal C Tran TH Zeier M Dohler B. Three-year outcomes following 1420 ABO-incompatible living-donor kidney transplants performed after ABO antibody reduction: results from 101 centers. Transplantation (2015) 99(2):400–4. 10.1097/TP.0000000000000312 25050471

[16] Biglarnia AR Nilsson B Nilsson T von Zur-Muhlen B Wagner M Berne C Prompt reversal of a severe complement activation by eculizumab in a patient undergoing intentional ABO-incompatible pancreas and kidney transplantation. Transpl Int (2011) 24(8):e61–6. 10.1111/j.1432-2277.2011.01290.x 21696455

[17] Takahashi K Saito K ABO-incompatible kidney transplantation. Transplant Rev (2013) 27(1):1–8. 10.1016/j.trre.2012.07.003 22902167

[18] Montgomery JR Berger JC Warren DS James NT Montgomery RA Segev DL. Outcomes of ABO-incompatible kidney transplantation in the United States. Transplantation (2012) 93(6):603–9. 10.1097/TP.0b013e318245b2af 22290268 PMC3299822

[19] Tydén G Kumlien G Fehrman I. Successful ABO-incompatible kidney transplantations without splenectomy using antigen-specific immunoadsorption and rituximab. Transplantation (2003) 76(4):730–1. 10.1097/01.TP.0000078622.43689.D4 12973118

[20] Genberg H Kumlien G Wennberg L Tyden G. The efficacy of antigen-specific immunoadsorption and rebound of anti-A/B antibodies in ABO-incompatible kidney transplantation. Nephrol Dial Transplant (2011) 26(7):2394–400. 10.1093/ndt/gfr237 21558429

[21] Zschiedrich S Janigen B Dimova D Neumann A Seidl M Hils S One hundred ABO-incompatible kidney transplantations between 2004 and 2014: a single-centre experience. Nephrol Dial Transplant (2016) 31(4):663–71. 10.1093/ndt/gfv388 26610596

[22] Barnett AN Manook M Nagendran M Kenchayikoppad S Vaughan R Dorling A Tailored desensitization strategies in ABO blood group antibody incompatible renal transplantation. Transpl Int (2014) 27(2):187–96. 10.1111/tri.12234 24188566

[23] Becker LE Siebert D Susal C Opelz G Leo A Waldherr R Outcomes following ABO-incompatible kidney transplantation performed after desensitization by nonantigen-specific immunoadsorption. Transplantation (2015) 99(11):2364–71. 10.1097/TP.0000000000000753 25989497

[24] Iwasaki K Miwa Y Ogawa H Yazaki S Iwamoto M Furusawa T Comparative study on signal transduction in endothelial cells after anti-a/b and human leukocyte antigen antibody reaction: implication of accommodation. Transplantation (2012) 93(4):390–7. 10.1097/TP.0b013e3182424df3 22222784

[25] Habicht A Broker V Blume C Lorenzen J Schiffer M Richter N Increase of infectious complications in ABO-incompatible kidney transplant recipients – a single centre experience. Nephrol Dial Transplant (2011) 26(12):4124–31. 10.1093/ndt/gfr215 21622990

[26] Lentine KL Axelrod D Klein C Simpkins C Xiao H Schnitzler MA Early clinical complications after ABO-incompatible live-donor kidney transplantation: a national study of medicare-insured recipients. Transplantation (2014) 98(1):54–65. 10.1097/TP.0000000000000029 24978035 PMC4411309

[27] Sharif A Alachkar N Bagnasco S Geetha D Gupta G Womer K Incidence and outcomes of BK virus allograft nephropathy among ABO- and HLA-incompatible kidney transplant recipients. Clin J Am Soc Nephrol (2012) 7(8):1320–7. 10.2215/CJN.00770112 22626962 PMC3408120

[28] Bentall A Neil D Sharif A Ball S ABO-incompatible kidney transplantation is a novel risk factor for BK nephropathy. Transplantation (2015) 99(2):e8–9. 10.1097/TP.0000000000000483 25651124

[29] Hall EC Engels EA Montgomery RA Segev DL. Cancer risk after ABO-incompatible living-donor kidney transplantation. Transplantation (2013) 96(5):476–9. 10.1097/TP.0b013e318299dc0e 23799426 PMC3759597

[30] Schaefer B Tonshoff B Schmidt J Golriz M Mehrabi A Gombos P Bleeding complications in pediatric ABO-incompatible kidney transplantation. Pediatr Nephrol (2013) 28(2):327–32. 10.1007/s00467-012-2302-x 22961637

[31] de Weerd AE van Agteren M Leebeek FW Ijzermans JN Weimar W Betjes MG. ABO-incompatible kidney transplant recipients have a higher bleeding risk after antigen-specific immunoadsorption. Transpl Int (2015) 28(1):25–33. 10.1111/tri.12412 25070762

[32] Kobayashi T Liu D Ogawa H Miwa Y Nagasaka T Maruyama S Removal of blood group A/B antigen in organs by ex vivo and in vivo administration of endo-beta-galactosidase (ABase) for ABO-incompatible transplantation. Transpl Immunol (2009) 20(3):132–8. 10.1016/j.trim.2008.09.007 18838121

[33] Roodnat JI van de Wetering J Claas FH Ijzermans J Weimar W. Persistently low transplantation rate of ABO blood type O and highly sensitised patients despite alternative transplantation programs. Transpl Int (2012) 25(9):987–93. 10.1111/j.1432-2277.2012.01526.x 22775425

