# Supplementary material for: ABO-Incompatible Kidney Transplantation
Source: Front Immunol. 2017 Mar 6;8:234. doi: 10.3389/fimmu.2017.00234 (PMC5338156; doi:10.3389/fimmu.2017.00234)
Supplement: Supplementary file 1 [file Table_1.PDF]

Supplemental Table S1: Selected studies on ABO incompatible kidney transplantation.

Author, Year	N	Years	Desensitization procedure	Successful Desensitization (%)	Follow-Up (Months)	Graft Survival (%)	AMR (%)
Montgomery et al., 2009	60	1999-2007	PP plus IVIg plus Spx (N=17) and/or Rtx (N=18) or no B-cell depleting therapy (N=28)	98.4	12<br>36<br>60	ABOi: 98.3*<br>ABOi: 92.9*<br>ABOi: 88.7*	ABOi: 16.7
Genberg et al., 2011	43	2001-2010	Selective IA plus IVIg plus Rtx<br>First study that uses anti-A/B antibody selective IA columns	95.6	54 (mean)	ABOi: 91	ABOi: 4.7
Flint et al., 2011<br>Melbourne, Australia	37	2005-2008	PP plus IVIg (until 2008)<br>Patients received conventional immunosuppression	n.a.	26 (median)<br>22 (median)	ABOi: 100<br>ABOc: 100, P=n.s.	ABOi: 5.4<br>ABOc: 0, P=n.s.
Fuchinoue et al., 2011<br>Tokyo, Japan	113	2002-2008	DFPP plus Spx (N=63) or Rtx (N=50)	n.a.	12<br><br><br>36	ABOi Spx: 96.8<br>ABOi Rtx: 100<br>ABOc: 99.2<br>ABOi Spx: 94.9<br>ABOi Rtx: 100<br>ABOc: 93.8	ABOi Spx: 15.9<br>ABOi Rtx: 4<br>ABOc: 2.5, P=0.651

					60	ABOi Spx: 90.3<br>ABOi Rtx: 100<br>ABOc: 88.4, P=0.726	
Montgomery et al., 2012<br>SRTR, US	738	1995-2010	n.a.	n.a.	12<br><br>36<br><br>120	ABOi: 94.1*<br>ABOc: 97.1*<br>ABOi: 89.6*<br>ABOc: 93.6*<br>ABOi: 72.9*<br>ABOc: 76.1*, P=0.001	n.a.
Takahashi and Saito, 2013<br>Japan, multicenter	1,427	2001-2010	n.a.	n.a.	12<br>36<br>108	ABOi: 96<br>ABOi: 93<br>ABOi: 83	n.a.
Barnett et al., 2014<br>London, UK	62	2005-2011	Selective IA or DFPP and/or PP plus IVIg (until April 2008) plus Rtx (until September 2010) or alemtuzumab (since October 2010)<br>Current protocol with DFPP (titer 1:16-1:64) or immunoadsorption (titer >1:64)	n.a.	12<br><br>36	ABOi: 92.9<br>ABOc: 98.1, P=0.060<br>ABOi: 90.3<br>ABOc: 96.2, P=0.072<br>Significant higher rate of death in ABOi compared to ABOc transplants (3 years: P=0.018)	ABOi: 4.8<br>ABOc: 1.2, P=0.124
Schiesser et al.,	71	2005-	Selective IA plus IVIg (at the beginning of	n.a.	12	ABOi: 100*	ABOi: 14.1

2015<br>Swiss, multicenter		2011	the study) plus Rtx<br>IA columns were reused after restoration		24<br>60	ABOi: 98.6*<br>ABOi: 97.2*	
Opelz et al., 2015<br>CTS, international registry	1,420	2005-2012	n.a.		36	ABOi: 89.9<br>ABOc: 90.1, P=0.44[#]<br>Significant higher rate of early infection-associated death in ABOi compared to ABOc transplants (1 year: P=0.037)[#]	n.a.
Becker et al., 2015<br>Heidelberg, Germany	34	2005-2013	Unselective IA plus 1x PP (since 2008) plus Rtx<br>First study that demonstrates equivalent anti-A/B antibody removal by unselective compared to selective IA	94.9	12<br>36<br>60	ABOi: 93.9<br>ABOc: 100<br>ABOi: 93.9<br>ABOc: 100<br>ABOi: 93.9<br>ABOc: 94.7, P=0.20	ABOi: 3<br>ABOc: 3, P=1.00
Zschiedrich, 2016<br>Freiburg, Germany	100	2004-2014	Selective IA or unselective IA plus 1x PP (since 2012) plus IVIg plus Rtx	n.a.	120	ABOi: 94*<br>ABOc: 88*, P=0.24	ABOi: 10<br>ABOc: 8, P=0.62
Bentall et al., 2016<br>UK, multicenter	100	2009-2012	Selective or unselective IA (N=55), DFPP or PP (N=31) plus Rtx (N=80) or alemtuzumab (N=4)	n.a.	12	ABOi: 94<br>ABOc: 97.6, P=0.04	ABOi: 4

Graft survival is given as overall graft survival or *death-censored graft survival; ABOc, ABO compatible, ABOi, ABO incompatible, AMR, antibody-mediated rejection; CTS, Collaborative Transplant Study; DFPP, double filtration plasmapheresis; IA, immunoadsorption; IVIg, intravenous immunoglobulins; n.a., not applicable; n.s., not significant; PP, plasmapheresis; Spx, splenectomy; SRTR, Scientific Registry of Transplant Recipients. [#]numbers are given for matched ABOc transplants.

References

Barnett, A.N., Manook, M., Nagendran, M., Kenchayikoppad, S., Vaughan, R., Dorling, A., et al. (2014). Tailored desensitization strategies in ABO blood group antibody incompatible renal transplantation. *Transpl Int* 27(2)**,** 187-196. doi: 10.1111/tri.12234.

Becker, L.E., Siebert, D., Susal, C., Opelz, G., Leo, A., Waldherr, R., et al. (2015). Outcomes Following ABO-Incompatible Kidney Transplantation Performed After Desensitization by Nonantigen-Specific Immunoadsorption. *Transplantation* 99(11)**,** 2364-2371. doi: 10.1097/TP.0000000000000753.

Bentall A., R Barnett A.N., Braitch M., Kessaris N., McKane W., Newstead C., et al. (2016). Clinical outcomes with ABO antibody titer variability in a multicenter study of ABO-incompatible kidney transplantation in the United Kingdom. *Transfusion*. doi: 10.1111/trf.13770. [Epub ahead of print]

Flint, S.M., Walker, R.G., Hogan, C., Haeusler, M.N., Robertson, A., Francis, D.M., et al. (2011). Successful ABO-incompatible kidney transplantation with antibody removal and standard immunosuppression. *Am J Transplant* 11(5)**,** 1016-1024. doi: 10.1111/j.1600-6143.2011.03464.x.

Fuchinoue S., Ishii Y., Sawada T., Murakami T., Iwadoh K., Sannomiya A., et al. (2011). The 5-year outcome of ABO-incompatible kidney transplantation with rituximab induction. *Transplantation* 91(8), 853-857. doi: 10.1097/TP.0b013e31820f08e8.

Genberg, H., Kumlien, G., Wennberg, L., and Tyden, G. (2011). The efficacy of antigen-specific immunoadsorption and rebound of anti-A/B antibodies in ABO-incompatible kidney transplantation. *Nephrol Dial Transplant* 26(7)**,** 2394-2400. doi: 10.1093/ndt/gfr237.

Montgomery, J.R., Berger, J.C., Warren, D.S., James, N.T., Montgomery, R.A., and Segev, D.L. (2012). Outcomes of ABO-incompatible kidney transplantation in the United States. *Transplantation* 93(6)**,** 603-609. doi: 10.1097/TP.0b013e318245b2af.

Montgomery R.A., Locke J.E., King K.E., Segev D.L., Warren D.S., Kraus E.S., et al. (2009). ABO incompatible renal transplantation: a paradigm ready for broad implementation. *Transplantation* 87(8), 1246-1255. doi: 10.1097/TP.0b013e31819f2024.

Opelz, G., Morath, C., Susal, C., Tran, T.H., Zeier, M., and Dohler, B. (2015). Three-year outcomes following 1420 ABO-incompatible living-donor kidney transplants performed after ABO antibody reduction: results from 101 centers. *Transplantation* 99(2)**,** 400-404. doi: 10.1097/TP.0000000000000312.

Schiesser M., Steinemann D.C., Hadaya K., Huynh-Do U., Eisenberger U., Binet I., et al., (2015). The Reuse of Immunoadsorption Columns in ABO-Incompatible Kidney Transplantation Is Efficient: The Swiss Experience. *Transplantation* 99(5), 1030-1035. doi: 10.1097/TP.0000000000000457.

Takahashi, K., and Saito, K. (2013). ABO-incompatible kidney transplantation. *Transplant Rev (Orlando)* 27(1)**,** 1-8. doi: 10.1016/j.trre.2012.07.003.

Zschiedrich, S., Janigen, B., Dimova, D., Neumann, A., Seidl, M., Hils, S., et al. (2016). One hundred ABO-incompatible kidney transplantations between 2004 and 2014: a single-centre experience. *Nephrol Dial Transplant* 31(4)**,** 663-671. doi: 10.1093/ndt/gfv388.
